# Slowed-Down Rehabilitation Following Percutaneous Repair of Achilles Tendon Rupture

**DOI:** 10.1177/10711007211038594

**Published:** 2021-09-28

**Authors:** Nicola Maffulli, Nikolaos Gougoulias, Gayle D. Maffulli, Francesco Oliva, Filippo Migliorini

**Affiliations:** 1 Department of Medicine, Surgery and Dentistry, University of Salerno, Baronissi (SA), Italy; 2 School of Pharmacy and Bioengineering, Keele University School of Medicine, Thornburrow Drive, Stoke on Trent, England, United Kingdom; 3 Queen Mary University of London, Barts and the London School of Medicine and Dentistry, Centre for Sports and Exercise Medicine, Mile End Hospital, London, England, United Kingdom; 4 General Hospital of Katerini, Greece; 5 Frimley Park Hospital, Frimley, Surrey, England, United Kingdom; 6 Wholelife Clinics, London, England, United Kingdom; 7 Department of Orthopaedics, University Clinic Aachen, RWTH Aachen University Clinic, Aachen, Germany

**Keywords:** Achilles tendon rupture, surgery, rehabilitation, slowed down

## Abstract

**Background:**

Following percutaneous repair of acute Achilles tendon (AT) ruptures, early postoperative weightbearing is advocated; however, it is debatable how aggressive rehabilitation should be. We compared the clinical and functional outcomes in 2 groups of patients who followed either our “traditional” or a “slowed down” rehabilitation after percutaneous surgical repair.

**Methods:**

Sixty patients were prospectively recruited to a slowed down (29 patients) or a traditional (31 patients) rehabilitation program. Both groups were allowed immediate weightbearing postoperatively; a removable brace with 5 heel wedges was applied at 2 weeks. In the slowed-down group, 1 wedge was removed after 4 weeks. Gradual removal of the boot took place after 4 wedges were kept for 4 weeks. In the traditional group, 1 wedge was removed every 2 weeks, with removal of the boot after 2 wedges had been kept for 2 weeks. The AT Resting Angle (ATRA) evaluated tendon elongation. Patient reported functional outcomes were assessed using the AT Rupture Score (ATRS). Calf circumference difference and the isometric plantarflexion strength of the gastro-soleus complex were evaluated.

**Results:**

At the 12-month follow-up, both ATRA and ATRS were more favorable in the slowed-down group. The isometric strength and the calf circumference were more similar to the contralateral leg in the slowed-down group than in the traditional one.

**Conclusion:**

Following percutaneous repair of acute Achilles tendon patients undergoing slowed down rehabilitation performed better than the traditional one. These conclusions must be considered within the limitations of the present study.

**Level of Evidence:**

Level II, prospective comparative study.

## Introduction

The management of acute Achilles tendon (AT) ruptures is debated. The process of tendon healing results in a fibrotic scar that impairs elasticity and may promote adherences,^17,23^ and impacts negatively on functional outcomes.^1,17,27^ Furthermore, the prolonged recovery causes absence from sports and reduces quality of life.^27,45^ In selected patients with AT rupture, surgery is recommended, and several surgical interventions have been proposed.^2,6,19,28,34,36^ Postoperative rehabilitation aims to prevent muscle atrophy and allow safe timely return to daily activities, sports, and other recreational activities. During the rehabilitation period, the AT undergoes profound structural remodeling and elongation.^25,41,44^ Therefore, the rehabilitation program must be strictly coordinated and well structured. We have been advocates of early weightbearing, early mobilization of the ankle with resistance exercises introduced at 4 weeks, and we have encouraged patients to discard relatively early the plantar cast at 2 weeks, and the brace, at 8 weeks, following percutaneous repair of Achilles tendon ruptures.^4,17,42,43,53^ This regimen, though successful, has been associated with an increased rate of postsurgical tendon elongation, regardless of whether absorbable or nonabsorbable suture material had been used for its repair,^59^ with functionally relevant decrease in strength of the gastro-soleus complex even with small amounts of elongation.^8^ Therefore, a prospective comparative study was conducted. Our hypothesis was that a “slowed down” rehabilitation program has advantages over our traditional protocol at 12 months in terms of calf circumference, isometric muscle strength, functional outcome, and tendon elongation after percutaneous repair of the AT.

## Material and Methods

### Study design

The present study was conducted according to the Consolidated Standards of Reporting Trials: the CONSORT statement.^40^ The present investigation was approved by our local ethics committee, and all patients gave their signed informed consent to participate. Patients were prospectively recruited at 2 different hospitals (the San Giovanni and Ruggi d’Aragona Hospital in Salerno and the Fucito Hospital in Mercato San Severino). Both were part of the same state-funded public health care system, but in different towns, with patients attending exclusively one or the other hospital. Patients were recruited between 2014 and 2017, being secondary or tertiary referrals to the senior author. To be eligible for inclusion, patients had to satisfy the following criteria: (1) primary AT repair, (2) complete the traditional or slowed down rehabilitation protocol, (3) age 18-50 years, and (4) ability to understand the nature of the treatment and the study. The exclusion criteria were (1) infections, (2) uncontrolled chronic disease, (3) pregnancy or lactation, (4) any blood abnormalities, (5) malignancy, (6) immunodeficiency, (7) previous or concurrent lower limb conditions that may have influenced the results of the study, (8) chronic Achilles tendinopathy, and (9) rerupture of the AT.

All procedures were performed by one experienced surgeon (N.M.) using a previously described percutaneous technique.^5^ Low-molecular-weight heparin thromboprophylaxis was used for 6 weeks. Postoperatively, a synthetic below-knee cast in maximum equinus, leaving the metatarsal heads free, was applied to all patients, regardless of the postoperative rehabilitation regimen. Full weightbearing mobilization on the metatarsal heads using elbow crutches was immediately allowed as tolerated. At the same time, active flexion and extension of the hallux, toes and knee, isometric exercises for the calf muscles, and straight leg raises were prescribed.

### Traditional Rehabilitation

The cast was removed after 2 weeks, an Aircast boot orthosis with 5 heel wedges (XP Walker, DJO Ltd, Guilford, England, United Kingdom) with the foot in maximum plantarflexion was applied, and full weightbearing in the Aircast boot was allowed. More demanding physiotherapy was started, including proprioception, ankle plantarflexion, inversion, and eversion exercises. Dorsiflexion and stretching exercises were not allowed.^35^ Beginning from the second until the eighth postoperative week, 1 heel wedge was removed every 2 weeks. At the eighth postoperative week, the Aircast boot was removed after the patients had been on 2 wedges for 2 weeks. Patients had normally regained a plantigrade ankle at that stage.^28,31,32^ Plyometric exercises were permitted 4 months after the index procedure, and patients were allowed to return to their normal activity, including sports, when they felt confident with doing so.^30^

### Slowed-Down Rehabilitation

Our slowed-down rehabilitation protocol is also based on the “early weightbearing” concept. However, removal of heel wedges to achieve a plantigrade foot starts 4 weeks later, and the Aircast boot is removed in a more gradual fashion (Table 1). From the eighth week postsurgery and over the course of 2 further weeks, patients were instructed to remove the brace increasing from 1 to 4 hours in the morning and again during the afternoon. At this stage, when not wearing the brace, patients were bearing weight on the operated leg, and were instructed to use a 15-mm heel wedge. At full removal of the brace, patients were required to use the 15-mm heel wedge for another month, and had normally regained a plantigrade ankle by that time. Only at that stage (after approximately 12 weeks) were they allowed to start eccentric exercises of the gastro-soleus complex. Plyometric exercises were permitted at 5 months after the index procedure, and patients were allowed to return to their normal activity, including sports, when they felt confident with doing so.^30^

**Table 1.** Schematic Comparison of the 2 Rehabilitation Protocols.

	Traditional Rehabilitation	Slowed-Down Rehabilitation
Equinus cast	0-2 wk	0-2 wk
Removable boot in maximum plantarflexion (5 wedges) and full weightbearing allowed	2 wk	2 wk
Proprioception, ankle plantarflexion, inversion, and eversion exercises, allowed	2 wk, unsupervised	2 wk, under physiotherapist’s supervision
Dorsiflexion and stretching exercises	Not allowed	Not allowed
Isometric gastro-soleus training	Allowed after 2 wk	Allowed after 2 wk
Removing boot wedges	One wedge removed every 2 wk, with removal of the boot after 2 wedges were kept for 2 wk	One wedge removed after 4 wk. Gradual removal of the boot after 4 wedged were kept for 4 wk
Crutches discarded	One crutch at approx. 2 wk (within 48 h after Aircast boot was applied) Both crutches at 4 wk	After 6 wk
Concentric exercises of the gastro-soleus complex	At 4 wk	After at least 6 wk
Plantarflexion, inversion, and eversion exercises against resistance	4 wk	After at least 6 wk
Aircast boot removed	8 wk—walking on plantigrade foot	12 wk (at 10 wk gradually discontinued the use of the brace by removing it increasingly from 1 to 4 hours in the morning and again during the afternoon, still using one 15-mm heel wedge out of boot). Patients used 1 heel wedge of 15 mm, from 12-16 wk.
Eccentric ankle exercises	8 wk	16 wk
Plyometric exercises	4 mo	5 mo
Normal daily activity, sports	Later than 4 mo, when feeling confident	Later than 5 mo, when feeling confident

### Outcomes of Interest

Age and gender of the patients recruited to the study were recorded at admission. At the 12-month follow-up, the patients were reviewed. To evaluate tendon elongation, the AT resting angle (ATRA)^6^ was measured. The angle was measured with the patient prone and the knee flexed to 90 degrees: the ATRA is the angle between the long axis of the fibula and the line from the tip of the fibula to the head of the fifth metatarsal.^7^ To assess patient-reported functional outcome, the AT rupture score (ATRS) was administered.^4^ Calf circumference was measured with the patient seated and the leg hanging over the side of an examination couch, with the knee flexed to 90 degrees. The examiner was careful to not compress the calf during the measurement process. The calf circumference was measured 15 cm below the medial palpable knee joint line using a standard metallic tape measure with 1-mm increments. This process was repeated twice and the average used for statistical analysis. The isometric plantarflexion strength of the gastro-soleus muscle complex was also evaluated at neutral position (0 degrees) as previously described.^30^ All these measurements were performed in the operated and compared to the healthy contralateral side.

### Statistical Analysis

With the ATRA as the main outcome with an effect size of 1.4 and alpha value of 0.05, it was estimated that 24 individuals per group would be required to adequately power the study.^16^ The IBM SPSS Software was used to calculate the arithmetic mean and standard deviation to each continuous measure. For the comparisons, the mean difference (MD) was used. The Shapiro-Wilk test was performed to investigate data distribution. For variables that satisfied the assumptions for parametric assumptions, the *t* test were performed, and the nonparametric Mann-Whitney *U* test for non-normally distributed variables. *P* values <.05 were considered statistically significant. *T* value, degrees of freedom, and 95% confidence intervals were calculated. The association between tendon elongation (ATRA), functional outcome (ATRS), gastro-soleus complex strength, and calf circumference difference were investigated. The Stata/MP (StataCorp, College Station, TX) was used. A linear model correlation analysis through the Pearson product-moment correlation coefficient (*r*) was used for parametric data, and the Spearman coefficient (ρ) for nonparametric variables. The Cauchy-Schwarz formula was used for inequality: +1 is considered as positive linear correlation, whereas −1 a negative one. Values of <0.3, 0.3 to 0.5, and >0.5 were considered to have small, medium, and strong correlation, respectively. The overall significance was performed through the χ^2^ test, with values of *P* <.05 considered statistically significant. A linear regression model was then performed for the significant correlations. Added-variable plots were also performed for each comparison.

## Results

### Recruitment Process

**Figure 1.**
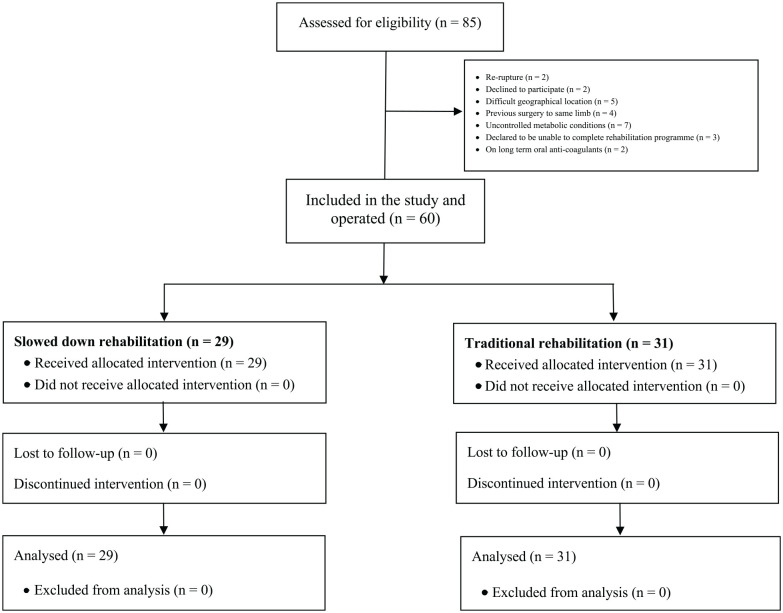
Flow diagram of the recruitment process.

A total of 85 patients were assessed for eligibility. Of them, 25 were not eligible because of rerupture (n = 2), declined to participate (n = 2), difficult geographical location (n = 5), previous surgery to same limb (n = 4), uncontrolled metabolic conditions (n = 7), declared to be unable to complete rehabilitation program (n = 3), and on long-term oral anticoagulants (n = 2). Ultimately, 60 patients were retained for the present analysis (Figure 1).

### Patient Demographic

All 60 patients practiced sport at the recreational level, with 1 to 3 sessions weekly. In the slowed-down group, 29 patients were enrolled, 19 (66%) of whom were male. The mean age was 39.2 ± 8.7 years, with a mean time from diagnosis to surgery of 4.1 ± 2.3 days. In the traditional rehabilitation group, 31 patients were enrolled, 24 (77%) of whom were male. The mean age was 35.8 ± 9.5 years, with mean time from diagnosis to surgery of 4.2 ± 2.7 days. Baseline comparability was found in terms of gender, age, and time from diagnosis to surgery between the 2 cohorts. Patient demographics are shown in Table 2.

**Table 2.** Patient Demographics.

Endpoint	Traditional (n = 31)	Slowed Down (n = 29)	*P*
Gender, male, %	77 (24 of 31)	66 (19 of 29)	.3
Age, y, mean ± SD	35.8 ± 9.5	39.2 ± 8.7	.2
Time from injury to surgery, d, mean ± SD	4.2 ± 2.7	4.1 ± 2.3	.9

### Outcomes of Interest

At the 12-month follow-up, the ATRA (+3.35; *P* < .0001) and ATRS (+1.87; *P* = .0007) differences were both more favorable in the slowed-down group. The evaluation of calf circumference showed better results in the slowed-down group, in which the circumference difference to the contralateral leg was smaller than in the traditional group (–0.50; *P* = .003). Similarly, the isometric strength also showed better results in the slowed-down group, in which the strength difference with the contralateral leg was smaller than in the traditional group (–14.42; *P* = .003). These results are shown in detail in Table 3. No patient experienced complications (infections, reruptures, loss of significant range of motion) during the follow-up in both groups.

**Table 3.** Comparison of ATRA, ATRS, Calf, and Isometric Strength Difference at 12-Month Follow-up.^a^

Endpoint	Traditional (n = 31)		Slowed Down (n = 29)		MD	95% CI	*t* Value	DF	*P*
	Mean	SD	Mean	SD					
ATRA, degrees	−4.48	3.31	−1.14	1.48	3.35	1.99-4.68	4.98	58	<.0001
ATRS, 0-100	90.06	1.82	91.93	2.22	1.87	0.82-2.91	3.57	58	.0007
Calf circumference, cm	1.32	0.65	0.81	0.60	−0.50	−0.83 to −0.18	−3.15	58	.003
Iso strength, N/m	47.94	21.04	33.52	13.81	−14.42	−23.67 to −5.14	−3.11	58	.003

Abbreviations: ATRA, Achilles tendon resting angle; ATRS, Achilles tendon rupture score; CI, confidence interval; DF, degrees of freedom; MD, mean difference.

a Data are presented as the difference between the operated leg and the contralateral.

### Analysis of Factors Related to the Outcome

**Figure 2.**
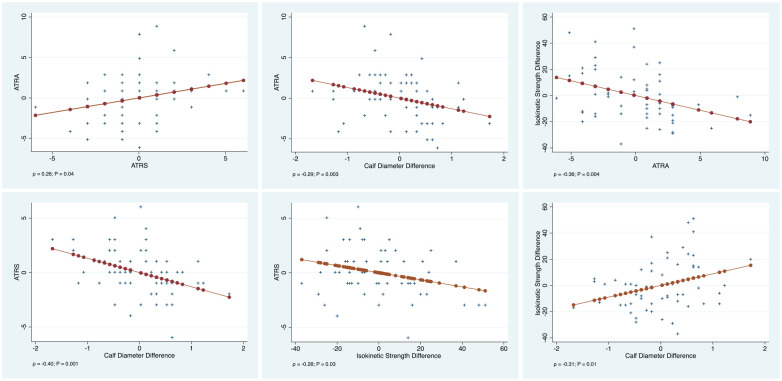
Added-variable plots of each linear regression.

Multivariate analysis showed an association between ATRA and ATRS ( r = 0.26; *P* = .04). Calf circumference difference was associated with ATRA ( r = −0.29; *P* = .003) and ATRS ( r = −0.40; *P* = .001). Isometric strength difference was associated with ATRA (*r* = −0.36; *P* = .004), ATRS ( r = −0.28; *P* = .03) and calf circumference difference ( r = −0.31; *P* = .01). Added-variable plots of each regression are shown in Figure 2.

## Discussion

The present study showed that a more cautious rehabilitation regimen resulted in a more favorable ATRA and ATRS compared to our standard protocol in patients undergoing primary percutaneous AT repair. We stress, however, that both regimens involved immediate postoperative weightbearing and early physiotherapy and mobilization. Calf circumference and isometric strength were more similar to the contralateral leg in patients who underwent such more cautious rehabilitation program. Moreover, there was evidence of a positive association between ATRA and ATRS, as well as between isometric strength and calf circumference. There was evidence of a negative association between calf circumference difference and increased difference in ATRA, as well as between isometric strength difference with ATRA.

AT ruptures lead to functional impairment and reduced muscle strength and endurance, representing a cause of sport retirement and long-term functional impairment.^19,33^ Management of these patients should aim to develop strategies increasing functional outcomes and avoiding possible complications.^10,11,13,19,29,37^ To our knowledge, this is the first study that compares 2 modes of rehabilitation, both involving early weightbearing and mobilization, but one retaining longer protection in a removable boot.

Rehabilitation takes place during a complex and only partially defined healing process, directed to restore tendon function. The poor vascularization and cellularity of tendons certainly influence the healing process,^3,20^ which can last more than 1 year. Unfortunately, knowledge of the process regarding tendon healing is limited. This results from various issues. First, most experimental tissue is harvested from acute tears.^46^ Moreover, there is significant variance between species in the healing pathways, and there is no appropriate animal model of actual Achilles tendon rupture for experimental studies.^8,9,50,54^ However, the tendon healing process is typically composed of 3 temporally partially overlapping phases: inflammation, proliferation, and remodeling.^21,57^ The inflammation phase starts directly after the acute rupture, with subsequent bleeding, platelet activation, and secretion of cytokines.^52,57^ This phase lasts a few days,^38^ and is followed by proliferation and migration of intrinsic resident cells (tenocytes and fibroblasts) and extrinsic cell populations (macrophages and other immune cells) to the injury site, with a complex secretion of cytokines such as insulinlike growth factor 1, platelet-derived growth factor, transforming growth factor–beta, and growth differentiation factor.^14,15,26,39,49,52,58^ This results in an early immature tissue formed by unorganized extracellular matrix with high content in fibronectin, proteoglycans, and collagen III.^18,23,24^ This stage lasts up to 2 months.^38^ Rehabilitation typically takes place during this phase. Therefore, patients must be followed by expert physicians and supervised by a trained physiotherapist.

ATRA is an indirect measure of the passive tension of AT from the ankle. Reduction of the ATRA between the operated and contralateral leg correlates with reduced tendon elongation. One centimeter of tendon elongation results in an increase of dorsiflexion of 10 degrees.^12^ Following AT rupture, a reduced plantar flexion strength up to 20% is expected.^22,47,55^ Moreover, AT rupture has been associated with reduced activity of the gastro-soleo complex compared with the uninjured side.^48^ These data suggest that reduced plantarflexion strength may be related to lengthening of the tendon during healing.^40,45^ Results from this study confirm this hypothesis. Furthermore, we identified an association between calf circumference difference, isometric muscle strength, ATRA and ATRS. Although these results have been found in a cohort of only 60 patients, they are statistically significant and reliable, and likely to be clinically relevant.

This study does not come without limitations. The relatively low number of subjects included in the study represents the most important limitation of the present study. Future larger investigations should address this point. The orthopaedic centers in which the patients were taken care of are a well-known excellence for the treatment of tendon conditions. The patients and the surgeon were not blinded to the procedure and to rehabilitation therapies, assessment, or cluster groups. This represents a possible source of detection bias. We are aware that this is not a randomized controlled trial. The lack of randomization involved in the allocation to treatment throws a barrier up to the inference about the efficacy of the rehabilitation regime itself. In this context, it is difficult to know how comparable the 2 groups at baseline are: despite the lack of marginal differences in age and gender, the relatively small group sizes may not provide sufficient power to detect differences if they exist. There are likely many unmeasured, and possibly unmeasurable, variables introducing selection biases that cannot be solved without a randomized trial or some other type of allocation scheme. Our power analysis determined that 24 individuals per group would be required to adequately power the study.^16^ We exceeded the number determined by the analysis to impart greater clinical relevance and generalizability to our investigation.^4-7,8^ However, we acknowledge that the high risk of selection bias and other unmeasured confounders may exert an influence in powering the sample size. Despite the weaknesses of the present investigation, our selection and recruitment process, our assessment criteria, and our follow-up were extremely rigorous, and performed in strict scientific fashion. Finally, the number of patients involved in the present investigation is comparable to is what reported by other studies on the management of this particular musculoskeletal ailment. The present study compared the outcomes in patients who followed 2 different rehabilitation protocols after percutaneous surgical repair.

A previous meta-analysis demonstrated that percutaneous AT repair compared to the open technique promote shorter surgical duration, reduce rate of infections, but higher incidence of sural nerve damage, whereas no difference was found in terms of rerupture, calf circumference, and range of motion.^56^ Biomechanical studies suggest that AT strength following reconstruction may be reduced following percutaneous procedures.^51^ Future studies should validate these results in using other AT repair strategies.

The most important strength of the present study was that all the surgeries were performed by the same fellowship-trained surgeon with long surgical experience and scientific expertise in the field of Achilles tendon surgery. All the surgical procedures were performed in the same fashion and with the same instruments, modalities, and materials. Also, all the patients were secondary and tertiary referrals, and they had already decided that they wanted to undergo surgical repair. In addition, no patient was lost to follow-up, a likely consequence of the preliminary screening process effected to enter the study, and of the motivation of both the patients and the research team. The outcomes of interest were not evaluated at admission, thus limiting the power to assess between-group baseline comparability. However, as all the patients suffered from an acute tear of the Achilles tendon, preoperative strength assessment of the calf muscle complex of the leg to be operated on is clearly ethically questionable. Data from a prior study, which used similar outcome measures, by our research group was used to estimate a priori sample size.^6^

## Conclusion

In our study population, slowed-down rehabilitation resulted in more favorable ATRA and ATRS compared to the traditional protocol in patients after primary percutaneous AT repair. Calf circumference and isometric strength were closer to the contralateral leg in the patients who underwent more cautious rehabilitation.

## Supplemental Material

sj-docx-1-fai-10.1177_10711007211038594 – Supplemental material for Slowed-Down Rehabilitation Following Percutaneous Repair of Achilles Tendon Rupture Click here for additional data file. Supplemental material, sj-docx-1-fai-10.1177_10711007211038594 for Slowed-Down Rehabilitation Following Percutaneous Repair of Achilles Tendon Rupture by Nicola Maffulli, Nikolaos Gougoulias, Gayle D. Maffulli, Francesco Oliva and Filippo Migliorini in Foot & Ankle International
