# Supplementary material for: Slowed-Down Rehabilitation Following Percutaneous Repair of Achilles Tendon Rupture
Source: Foot Ankle Int. 2021 Sep 28;43(2):244–52. doi: 10.1177/10711007211038594 (PMC8841642; doi:10.1177/10711007211038594)
Supplement: sj-docx-1-fai-10.1177_10711007211038594 – Supplemental material for Slowed-Down Rehabilitation Following Percutaneous Repair of Achilles Tendon Rupture [file sj-docx-1-fai-10.1177_10711007211038594.docx]

FAI-21-0245.R1 – Reported Author Disclosures

Slowed down rehabilitation following percutaneous repair of Achilles tendon rupture

The author(s) declared no potential conflicts of interest with respect to the research, authorship, and/or publication of this article. ICMJE forms for all authors are available online.
